# Microbial metabolites in chemotherapy-induced cognitive impairment: from mechanistic associations to pharmacological target validation

**DOI:** 10.3389/fphar.2026.1968616

**Published:** 2026-10-30

**Authors:** Yuanyuan Tian, Wentao Li, Juyue Zhou, Jianchun Yu

**Affiliations:** 1 First Teaching Hospital of Tianjin University of Traditional Chinese Medicine, Tianjin, China; 2 National Clinical Research Center for Chinese Medicine, Tianjin, China; 3 Graduate School, Tianjin University of Traditional Chinese Medicine, Tianjin, China; 4 Department of Oncology, First Teaching Hospital of Tianjin University of Traditional Chinese Medicine, Tianjin, China

**Keywords:** cancer therapy, chemotherapy-induced cognitive impairment, gut microbiota, gut-brain axis, microbial metabolites, neuroinflammation, pharmacological intervention

## Abstract

Chemotherapy-induced cognitive impairment (CICI) encompasses heterogeneous changes in memory, attention, executive function and processing speed. Microbial metabolites offer experimentally tractable candidates because their exposure, dose–response relationships and host-target engagement can be measured. This review evaluates short-chain fatty acids (SCFAs), microbial indoles and host kynurenine metabolism, and microbially modified bile acids, while considering microbial inflammatory products as distinct modifiers of the intestinal–brain environment. Evidence is assessed against five criteria: CICI specificity, temporal sequence, selective causal manipulation, host-target engagement and oncological compatibility. Clinical microbiome associations and probiotic intervention findings support further investigation, but do not establish mediation by an individual metabolite. Selected chemotherapy models provide cognitive and molecular intervention evidence, although its strength varies by agent, metabolic pathway and behavioral endpoint. Changes in metabolite concentrations may reflect altered diet, epithelial absorption, host metabolism or clearance as well as microbial production. Translation therefore requires regimen-specific longitudinal sampling, compartment- appropriate exposure measurements, selective pathway perturbation and domain-specific cognitive assessment with controls for sickness and motor impairment. Cognitive protection must also be evaluated alongside chemotherapy exposure, tumor control and infectious safety. This framework distinguishes biomarkers of treatment-associated injury from causal mediators and identifies the evidence needed to develop metabolite-guided pharmacological interventions.

## 1 Introduction

Chemotherapy-induced cognitive impairment (CICI) includes changes in learning and memory, attention, executive function and processing speed associated with cytotoxic treatment. It forms part of the broader spectrum of cancer-related cognitive impairment, in which disease, other treatments and patient-related factors may also contribute. Variation across regimens and cognitive domains makes precise phenotyping essential: subjective difficulties and objective test performance provide complementary information, and neither should be treated as a uniform outcome (Wefel et al., 2011; Otto-Dobos et al., 2024).

The intestinal environment is a plausible contributor to this heterogeneity, but clinical findings currently support association more strongly than metabolite-specific causality. In a longitudinal study of 77 patients with breast cancer, reduced microbial diversity was associated with declining objective cognition, whereas subjective cognitive changes showed a different relationship with the measured biological signals (Otto-Dobos et al., 2024). A pilot study in 20 women associated single pretreatment stool and saliva profiles with self-reported cognitive impairment assessed using the Functional Assessment of Cancer Therapy–Cognitive Function (FACT-Cog) questionnaire during the first two chemotherapy cycles (Cross et al., 2026). Its two- feature model underwent five-fold cross-validation and permutation testing, but lacked independent external validation and longitudinal omic measurements. Together, these observational studies nominate candidate risk signals without establishing that a particular microbial metabolite mediates CICI.

Small-molecule metabolites offer a practical route from these associations to pharmacological testing. A defined compound can be chemically quantified, administered at a specified dose and evaluated for exposure–response relationships and engagement of a host target. Its site of action can also be investigated in intestinal, immune, vascular or central compartments. Metabolite intervention experiments illustrate this tractability, while highlighting that molecular protection and behavioral benefit require separate validation (Ren et al., 2026). These advantages depend on achievable tissue exposure and selective mechanisms; a circulating concentration alone does not establish either.

The central distinction is between a biomarker of exposure or injury and a causal mediator. A metabolite may track epithelial damage, altered food intake or hepatic clearance without contributing to cognitive dysfunction. A mediator must instead influence the cognitive outcome through a demonstrable host pathway, which may act locally in the intestine or through peripheral or central signaling. We therefore evaluate short-chain fatty acids (SCFAs), microbial indoles and host kynurenine metabolism, and microbially modified bile acids against five criteria: CICI specificity, temporal sequence, selective causal manipulation, host-target engagement and compatibility with anticancer treatment. Microbial structural products are considered separately as inflammatory modifiers. This framework guides assessment of both microbiota-directed interventions and host-targeted pharmacology while keeping cognitive benefit and tumor control within the same validation strategy.

We used targeted searches for PubMed-indexed literature and citation checking to update this narrative review on 19 October 2026. Searches used combinations of concepts concerning chemotherapy, cognitive impairment, the gut microbiota, SCFAs, tryptophan/indole/kynurenine metabolism, bile acids, intestinal permeability and lipopolysaccharide (LPS). Clinical studies and chemotherapy-exposed animal models informed CICI-specific judgments, with cognitive endpoints distinguished from intestinal, affective and molecular outcomes; studies in other disease models and cellular systems provided mechanistic context.

## 2 Chemotherapy reshapes the intestinal metabolic environment

Chemotherapy reshapes the intestinal metabolic environment through overlapping but nonuniform mechanisms. Drug class, individual agent, dose, formulation and treatment schedule influence the balance between epithelial injury, inflammatory signaling, altered nutrient supply and host metabolic handling. A comparison of paclitaxel, cyclophosphamide, cisplatin and doxorubicin in female mice illustrates this distinction: circulating lipopolysaccharide-binding protein (LBP) increased shortly after all four regimens, whereas the persistence and pattern of microbial, inflammatory and metabolic changes differed (Valentine et al., 2026). These observations support shared acute responses alongside regimen-dependent effects; the study assessed fatigue- and anxiety-related behavior rather than cognition.

Platinum compounds require distinction at the agent and tissue levels. Although cisplatin and oxaliplatin share DNA-damaging actions, their associated metabolic findings should not be treated as a uniform platinum signature. Cisplatin-associated cerebral kynurenic acid (KYNA) elevation was examined alongside recognition-memory impairment, whereas oxaliplatin-associated myeloid indoleamine 2,3-dioxygenase 1 (IDO1) induction and kynurenine accumulation were investigated primarily in relation to intestinal toxicity (Dorcas et al., 2024; Xie et al., 2026). These studies nominate different mechanistic questions and cannot establish that the same metabolic pathway mediates cognitive effects across platinum regimens. Peripheral neurotoxicity also requires attention when interpreting motor-dependent behavioral outcomes.

For taxanes, the microtubule-directed drug action must be considered alongside changes in intestinal and hepatobiliary physiology. In paclitaxel-treated mice, reduced secondary bile acid formation occurred together with diminished ileal absorptive capacity and altered hepatic expression of bile acid synthesis and transport genes (Loman et al., 2024). LPS exposure reproduced several hepatic transcriptional changes in isolated hepatocytes more closely than direct paclitaxel exposure, indicating an inflammatory contribution to disrupted bile acid handling. Thus, an altered bile acid pool cannot be attributed solely to microbial enzyme loss or direct drug toxicity. These findings require independent confirmation for docetaxel and other schedules, and transporter transcript changes alone do not establish altered chemotherapy clearance.

Antimetabolite-associated mucositis introduces another route to metabolic disruption. In a 5- fluorouracil (5-FU) model, early crypt-cell apoptosis preceded later dysbiosis and inflammatory changes, supporting a sequence in which epithelial injury helps initiate ecological disruption (Hamouda et al., 2017). Changes in absorption, transit and luminal substrate availability may consequently alter measured metabolites without indicating a primary defect in microbial biosynthesis. Findings from a particular injected 5- FU schedule should therefore not be assumed to describe oral capecitabine-containing regimens, for which route, exposure pattern and combination treatment require separate evaluation.

Reduced food intake is a mechanistic confounder as well as a consequence of treatment. When chemotherapy induces hypophagia, animal studies should incorporate vehicle-treated *ad libitum* controls, chemotherapy-treated animals, and vehicle-treated pair-fed controls receiving the same diet in quantities matched to the chemotherapy group’s measured intake. Feeding timing and prolonged fasting should also be considered. Comparing pair-fed with *ad libitum* controls estimates effects of reduced intake, whereas comparison with chemotherapy-treated animals identifies changes not explained by intake alone. A methotrexate study demonstrated intestinal injury and metabolic disturbances beyond those observed in pair-fed controls, illustrating the value of this design (Boukhettala et al., 2009). Nevertheless, pair-feeding does not equalize nutrient absorption or exclude indirect inflammatory effects, so residual differences should not automatically be labeled direct drug-induced dysbiosis.

Clinical studies should record actual dietary intake, gastrointestinal symptoms, antibiotics, antiemetics, corticosteroids, acid-suppressive therapy and hepatic or renal dysfunction alongside the complete chemotherapy regimen. Sampling before treatment, during defined cycles and after recovery is needed to distinguish transient toxicity from persistent metabolic change. In combination regimens, a signal should initially be attributed to the tested regimen unless component-specific evidence is available. Across the pathways evaluated below, metabolic alterations should therefore be interpreted as candidate agent- or regimen-dependent mechanisms, with shared stress responses identified through comparative evidence rather than assumed.

## 3 Microbial metabolites linking intestinal dysbiosis to cognitive impairment

The evidence differs sharply in its proximity to CICI. Longitudinal metabolomics in patients and interventions with cognitive endpoints are most informative; tumor-bearing chemotherapy models can support causality when exposure, metabolites and cognition are examined together. Findings from aging, neurodegeneration or non-cancer inflammation provide mechanistic context but should not be presented as direct CICI evidence. Cognitive phenotyping also matters (Wefel et al., 2011). Patient-reported difficulty and objective test performance capture overlapping but non-identical outcomes, and both can be affected by fatigue, pain, sleep disturbance, anxiety and depression (Mihuta et al., 2016; Ahles and Root, 2018). Repeated, domain-specific assessment is therefore preferable to a single global screen when linking a metabolic change to CICI (Wefel et al., 2011). Figure 1 summarizes candidate sites of exposure and host-target engagement across the intestinal–immune–brain axis.

**FIGURE 1:**
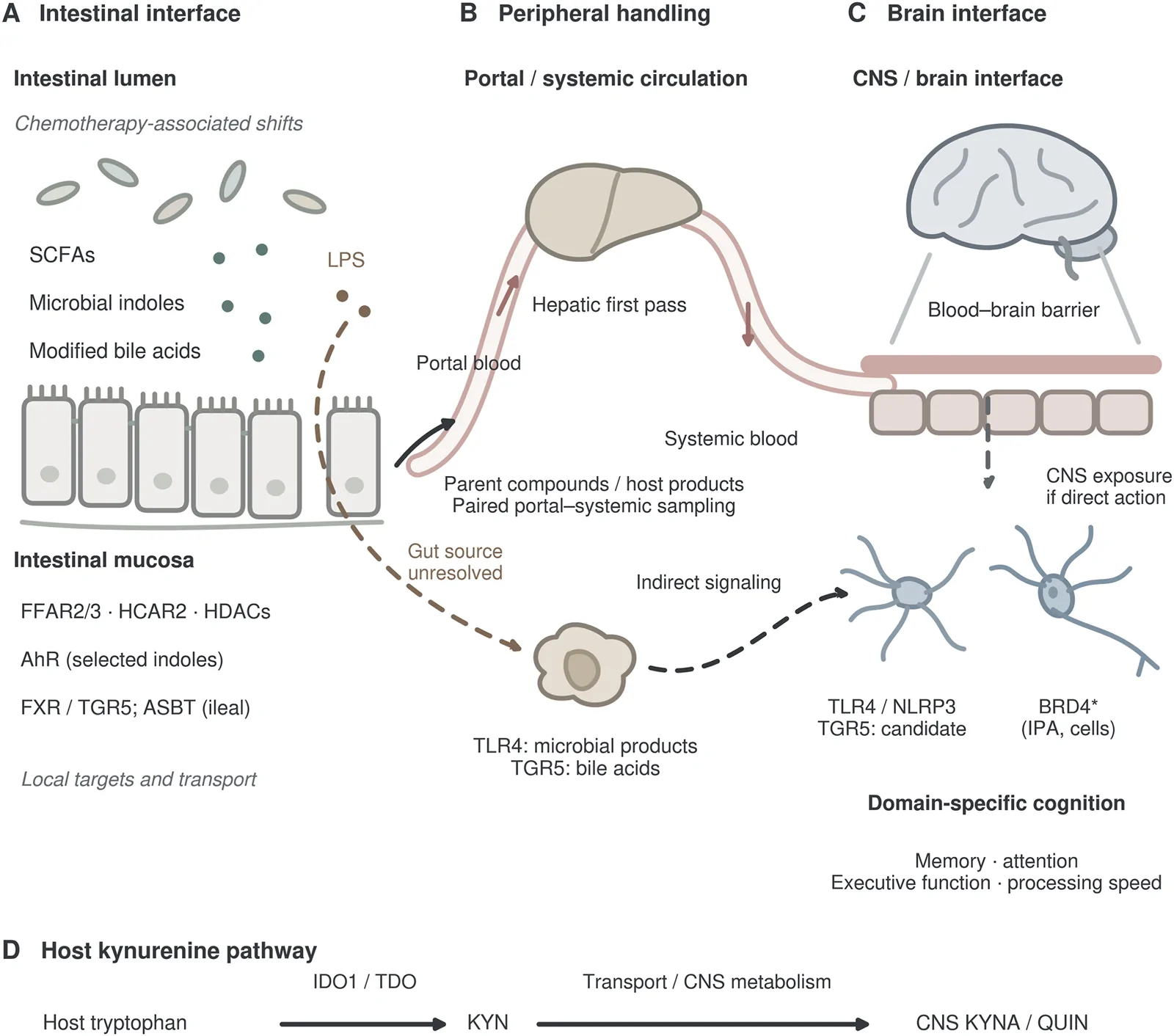
Candidate sites of exposure and target engagement across the intestinal–immune–brain axis in CICI. **(A)** Luminal SCFAs, microbial indoles and microbially modified bile acids are shown separately from LPS, a microbial structural product. Mucosal mechanisms include FFAR2/3, HCAR2 and HDAC-related effects, ligand-specific AhR signaling, and FXR/TGR5; ASBT denotes active ileal bile acid reabsorption. **(B)** Absorption, hepatic first-pass handling, host metabolism and systemic clearance shape measured exposure. Paired portal and systemic sampling helps distinguish these processes. **(C)** The neurovascular inset locates peripheral, endothelial and potential central signaling, including TLR4/NLRP3 and candidate neural TGR5 pathways. Direct central mechanisms require relevant CNS exposure. The asterisk marks cellular IPA–BRD4 findings from the arsenic trioxide study; *in vivo* BRD4 dependence of cognitive benefit remains to be tested. Cognitive domains denote assessment outcomes. **(D)** Host IDO1/TDO–kynurenine metabolism is shown separately from luminal microbial production. Solid arrows summarize biological handling or host-pathway relationships; dashed arrows indicate candidate source or signaling connections requiring CICI-specific verification. The five validation criteria are CICI specificity, temporal sequence, selective causal manipulation, compartment-specific host- target engagement and oncological compatibility under the intended chemotherapy regimen. Abbreviations: AhR, aryl hydrocarbon receptor; ASBT, apical sodium-dependent bile acid transporter; BRD4, bromodomain-containing protein 4; CICI, chemotherapy-induced cognitive impairment; CNS, central nervous system; FFAR2/3, free fatty acid receptors 2/3; FXR, farnesoid X receptor; HCAR2, hydroxycarboxylic acid receptor 2; HDAC, histone deacetylase; IDO1, indoleamine 2,3-dioxygenase 1; IPA, indole-3-propionic acid; KYN, kynurenine; KYNA, kynurenic acid; LPS, lipopolysaccharide; NLRP3, NLR family pyrin domain containing 3; QUIN, quinolinic acid; SCFA, short-chain fatty acid; TDO, tryptophan 2,3-dioxygenase; TGR5, Takeda G protein-coupled receptor 5; TLR4, Toll-like receptor 4.

### 3.1 Short-chain fatty acids and neuroimmune homeostasis

Acetate, propionate and butyrate connect microbial carbohydrate fermentation with host metabolism and immune regulation. Their pharmacological actions include signaling through free fatty acid receptors 2 and 3 (FFAR2/3), activation of hydroxycarboxylic acid receptor 2 (HCAR2) by butyrate, and concentration-dependent inhibition of histone deacetylases (HDACs) (Dalile et al., 2019; Silva et al., 2020). These mechanisms should be distinguished because receptor activation, epithelial fuel provision and intracellular HDAC inhibition require different exposure conditions. Non-chemotherapy experiments linking microbial metabolites to blood–brain barrier integrity and SCFAs to microglial homeostasis provide mechanistic support, but do not establish cognitive efficacy during chemotherapy (Braniste et al., 2014; Erny et al., 2015).

CICI specificity. Chemotherapy studies support intestinal and neuroimmune effects more directly than cognitive rescue. Oral sodium butyrate ameliorated intestinal dysfunction and anxiety- and depression-related behaviors in paclitaxel-treated mice; these outcomes should not be equated with improved learning or memory (Cristiano et al., 2022). In a 5-FU model, a high-fiber diet increased cecal propionate and reduced glial fibrillary acidic protein (GFAP) staining in selected brain regions, but cognitive performance was not assessed (Cross et al., 2024). Thus, these studies identify chemotherapy-responsive pathways without demonstrating that a specific SCFA mediates CICI.

Temporal sequence. Neither study establishes that an SCFA deficit precedes objective cognitive decline. Reduced fermentable substrate intake, intestinal transit changes, stool water content and altered absorption can influence measured concentrations independently of microbial synthetic capacity. Longitudinal studies should therefore combine dietary and gastrointestinal assessments with repeated SCFA measurements and cognitive testing. Fecal concentrations represent the balance of production, utilization and excretion, rather than a direct measure of systemic exposure.

Causal manipulation. Defined butyrate administration provides a more specific intervention than dietary enrichment, but evidence of intestinal or affective benefit still requires extension to cognitive endpoints. Conversely, a fiber intervention changes multiple microbial functions and metabolites, so an association between propionate and reduced glial staining does not demonstrate propionate mediation. Selective supplementation, dose response assessment and interruption of the proposed signaling pathway should be combined with cognitive tests that control for motor impairment and sickness behavior.

Host-target engagement. Validation should identify whether the relevant action occurs in intestinal epithelial cells, peripheral immune cells, vascular endothelium or the central nervous system. Compartment-appropriate exposure measurements should be linked to receptor-dependent signaling or HDAC-related pharmacodynamic changes. Receptor blockade or conditional genetic disruption can test pathway necessity, whereas histone acetylation or reduced inflammatory markers alone cannot identify a unique SCFA mechanism. Direct central action requires evidence of relevant central exposure; indirect intestinal or immune mechanisms do not require the parent metabolite to enter the brain.

Oncological compatibility. The cited studies do not establish a package of SCFA-mediated cognitive benefit, defined target engagement and preserved tumor control. Tumor-bearing chemotherapy models should assess these outcomes together, including drug exposure and gastrointestinal tolerability. SCFAs therefore remain pharmacologically tractable candidates with chemotherapy-related intestinal and neuroimmune evidence, while CICI-specific temporal, causal and oncological validation remains incomplete.

### 3.2 Tryptophan metabolism at the interface of microbial and host signaling

Tryptophan metabolism links microbial activity to host immune and neural signaling, but its branches require separate attribution. Host IDO1 and tryptophan 2,3-dioxygenase (TDO) initiate kynurenine production, whereas intestinal microorganisms generate indole derivatives (Liu et al., 2022). Peripheral kynurenine can enter the brain more readily than KYNA or quinolinic acid (QUIN), making circulating concentrations of these downstream products imperfect indicators of central exposure (Fukui et al., 1991). QUIN can promote N-methyl-D-aspartate receptor-mediated excitotoxicity, whereas KYNA antagonizes excitatory signaling and may disrupt cognition when excessive (Kennedy et al., 2017). Consequently, an increase in pathway activity does not identify the responsible metabolite, its tissue of origin, or its functional effect.

Microbial regulation of host kynurenine metabolism may operate indirectly through changes in tryptophan availability and immune signaling, so chemotherapy-associated IDO1 induction should not automatically be assigned an intestinal microbial origin (Kennedy et al., 2017). Downstream branching is equally important: kynurenine aminotransferases generate KYNA, whereas the kynurenine 3-monooxygenase-containing branch supplies precursors for QUIN (Kennedy et al., 2017). These routes can produce different effects on excitatory neurotransmission. Upstream IDO1 inhibition, branch-specific enzyme modulation and antioxidant treatment therefore test distinct mechanisms; benefit from one approach cannot establish the cognitive efficacy of another.

CICI specificity. Evidence differs between chemotherapy agents and metabolic branches. In cisplatin-treated female rats, increased cerebral KYNA accompanied impaired long-term novel-object recognition; N-acetylcysteine attenuated both abnormalities, although its antioxidant effects prevent attribution to selective kynurenine-pathway modulation (Dorcas et al., 2024). By contrast, an oxaliplatin study linked myeloid-cell IDO1 induction and kynurenine accumulation to intestinal toxicity without establishing cognitive consequences (Xie et al., 2026). For microbial indoles, Ren et al. (2026) reported that oral indole-3-propionic acid (IPA) improved Morris water maze performance and hippocampal injury in arsenic trioxide-treated mice. IPA was administered daily by gavage at 20, 50 or 100 mg/kg during arsenic trioxide treatment, with the clearest water-maze benefit at 50 mg/kg/day. This provides direct treatment-specific evidence, but concurrent changes in locomotor activity warrant caution when interpreting behavioral specificity, and applicability to other chemotherapy regimens remains untested.

Temporal sequence. These findings do not establish a longitudinal sequence in patients in which a defined tryptophan-metabolite abnormality precedes objective cognitive decline. Ren et al. (2026) reported reduced plasma IPA in arsenic trioxide-treated patients, but this observation does not itself demonstrate a metabolite–cognition relationship. Serial measurements before treatment, during exposure, and after treatment should distinguish early metabolic changes from consequences of intestinal injury, reduced food intake, or established neurotoxicity. Kynurenine-to-tryptophan ratios may aid pathway characterization but should not be interpreted as tissue-specific IDO1 activity without accounting for TDO, substrate availability, and clearance.

Causal manipulation. IPA supplementation provides intervention evidence beyond a compositional microbiome association (Ren et al., 2026). Nevertheless, benefit from an administered metabolite does not by itself establish that loss of endogenous microbial production caused the impairment. Selective manipulation of IPA-producing capacity, followed by metabolite rescue, would strengthen that causal link. Likewise, the cisplatin findings support investigation of KYNA but do not establish that selective IDO1 inhibition rescues cognition (Dorcas et al., 2024). Experiments should separate prevention during chemotherapy from reversal of established impairment and include controls for activity, sickness, and nutritional status.

Host-target engagement. Microbial indoles should not be assigned a common receptor mechanism solely because they share a metabolic origin. Aryl hydrocarbon receptor (AhR)-dependent regulation of astrocyte inflammation by microbial tryptophan metabolites has been demonstrated outside CICI (Rothhammer et al., 2016). In the arsenic trioxide study, IPA preserved bromodomain-containing protein 4 (BRD4), histone H4 acetylation and phosphofructokinase expression. A cellular thermal shift assay (CETSA) showed BRD4 thermal stabilization in HT22 cells treated with 100 μM IPA for 24 h, supporting cellular target engagement, while BRD4 knockdown attenuated IPA-associated molecular protection (Ren et al., 2026). Dose- linked brain IPA concentrations were not reported, and BRD4 dependence of the behavioral benefit *in vivo* remains untested.

Oncological compatibility. IDO1 is particularly relevant because tryptophan catabolism can support tumor immune escape while supplying precursors for neuroactive kynurenines (Uyttenhove et al., 2003). Its inhibition therefore offers a testable opportunity to combine cognitive protection with improved antitumor immunity, rather than an established dual benefit. The negative ECHO-301/KEYNOTE-252 trial of epacadostat plus pembrolizumab in melanoma illustrates that mechanistic rationale does not guarantee clinical antitumor efficacy; that trial did not test CICI (Long et al., 2019). The IPA study likewise does not establish preservation of anticancer efficacy during cognitive protection. Overall, tryptophan metabolism has treatment-specific cognitive and intervention evidence, but longitudinal clinical validation, *in vivo* target dependence, and concurrent demonstration of tumor control remain incomplete.

### 3.3 Microbially modified bile acids as an exploratory pathway

Microbial deconjugation and subsequent 7α-dehydroxylation reshape the host-derived bile acid pool, including conversion of cholic acid to deoxycholic acid and chenodeoxycholic acid to lithocholic acid (Wahlström et al., 2016; Ridlon et al., 2016). Farnesoid X receptor (FXR/*NR1H4*) is a nuclear receptor linking bile acid homeostasis with intestinal defense; Takeda G protein-coupled receptor 5 (TGR5/*GPBAR1*) mediates membrane-initiated signaling. Ligands have distinct pharmacology: chenodeoxycholic acid, a primary bile acid, activates FXR, whereas the secondary bile acids deoxycholic and lithocholic acids can activate TGR5 (Wahlström et al., 2016; Ridlon et al., 2016). In non-chemotherapy models, intestinal FXR activation induces enteroprotective genes and limits bacterial overgrowth and mucosal injury, while TGR5-dependent cyclic adenosine monophosphate (cAMP)–protein kinase A (PKA) signaling in macrophages restrains activation of the NLR family pyrin domain containing 3 (NLRP3) inflammasome (Inagaki et al., 2006; Guo et al., 2016). These findings support intestinal and immune mechanisms without establishing cognitive benefit during chemotherapy. Ligand composition, tissue exposure and cell-specific responses must therefore be evaluated separately; a lower total or secondary bile acid concentration does not by itself establish reduced signaling through either receptor.

CICI specificity. In paclitaxel-treated mice, Loman et al. (2024) reported altered enterohepatic bile acid metabolism, reduced secondary bile acid formation, and diminished ileal absorptive surface area and expression of *Slc10a2* and *Fgf15*. The apical sodium-dependent bile acid transporter (ASBT), encoded by *SLC10A2*, mediates active bile acid uptake in the terminal ileum. Reduced epithelial uptake can alter colonic bile acid availability independently of a primary change in microbial enzymatic capacity; host transport and microbial conversion therefore require joint assessment. Cognitive outcomes were not assessed, so the study documents a paclitaxel-associated enterohepatic disturbance that warrants testing in CICI.

Temporal sequence and causal manipulation. The cited chemotherapy study does not establish that a defined bile acid alteration precedes cognitive decline, or that selective correction of that alteration prevents or reverses CICI. In a non-chemotherapy setting, Ren et al. (2024) linked enhanced intestinal bile acid absorption to age-related cognitive impairment and demonstrated benefits of interventions targeting intestinal bile acid handling. This provides mechanistic support for an absorption–brain relationship, but its direction differs from the impaired ileal absorption reported after paclitaxel. It therefore cannot justify ASBT inhibition as a general CICI treatment. Longitudinal, regimen-specific studies should first establish the direction of bile acid disruption and its relationship to objective cognition, followed by selective ligand, transporter, or receptor manipulation with appropriate behavioral controls.

Host-target engagement. Evidence linking bile acid receptor modulation to cognition also comes from non-CICI models. For example, in a rat sepsis model, the TGR5 agonist INT-777 improved cognitive outcomes and attenuated neuroinflammation, accompanied by changes in cAMP–PKA signaling and phosphorylation of cAMP response element-binding protein (CREB) (Jin et al., 2021). These findings provide non-CICI pharmacological support, while the involvement of endogenous secondary bile acids during chemotherapy remains unresolved. CICI experiments should distinguish intestinal or peripheral immune signaling from direct central actions. FXR-responsive transcription or TGR5-dependent signaling should be measured in the proposed target tissue and paired with receptor-specific loss-of- function tests. A proposed direct central mechanism additionally requires evidence of relevant central exposure; an intestinal mechanism does not require the parent bile acid to enter the brain. Neither circulating bile acid concentrations nor receptor expression alone demonstrates functional target engagement.

Oncological compatibility. The studies discussed above do not establish that manipulating bile acids, ASBT, FXR, or TGR5 improves cognition while preserving chemotherapy efficacy. Validation therefore requires tumor-bearing models that assess cognitive outcomes alongside chemotherapy exposure, tumor response, intestinal injury, and hepatic function. Interventions should correct a demonstrated, regimen-specific abnormality rather than assume that increasing secondary bile acids or suppressing their absorption is universally beneficial. Under the five-criterion framework, bile acids remain an exploratory CICI pathway: chemotherapy-associated metabolic disruption is documented, whereas temporal linkage to cognition, selective causal rescue, compartment-specific target engagement, and oncological compatibility remain to be established.

### 3.4 Microbial inflammatory products as modifiers of metabolite–brain signaling

LPS is a microbial structural product rather than a small-molecule metabolite. Its relevance here is as a potential inflammatory modifier of metabolite–brain signaling. The proposed sequence links epithelial injury to increased microbial-product exposure, peripheral immune or endothelial responses, altered neuroimmune signaling and cognitive dysfunction. Each connection requires evidence: intestinal histological injury, systemic inflammation and cognitive impairment occurring together do not establish the complete causal chain.

CICI specificity. In paclitaxel-treated mice, altered colonic structure and microbiota accompanied increased circulating LBP and impaired contextual memory, supporting an association between intestinal disturbance and neurobehavioral changes (Loman et al., 2019). Separate microbiota-manipulation experiments assessed inflammatory responses, locomotion and anxiety-related behavior, without demonstrating memory rescue (Grant et al., 2021). PLX5622, an inhibitor of colony-stimulating factor 1 receptor (CSF1R), attenuated hippocampal inflammatory gene expression and contextual fear-conditioning deficits when administered from 5 days before chemotherapy and continued throughout the experiment (Grant et al., 2023). Potential peripheral effects of CSF1R inhibition limit attribution of this benefit exclusively to microglia. These findings support distinct components of the proposed pathway but do not identify gut-derived LPS as the signal connecting them.

Temporal sequence. Repeated sampling should establish whether functional barrier disruption precedes increased microbial-product exposure, whether exposure precedes neuroimmune changes, and whether these changes predict subsequent cognitive decline. Sampling only after cognitive impairment has developed cannot distinguish a mediator from a consequence of treatment-related injury. Timing relative to chemotherapy, infection, neutropenia and supportive medications is particularly important because inflammatory responses can be transient.

Causal manipulation. Stronger evidence would combine an intervention that selectively limits intestinal microbial-product exposure with exposure measurements, cognitive outcomes and disruption of the proposed downstream pathway. Broad antibiotics and microbiota transfer affect multiple metabolites and host processes, while systemic LPS injection does not reproduce the route or kinetics of endogenous translocation. In a cisplatin model, global *Tlr4* or *Nlrp3* deletion and intracerebroventricular administration of TAK-242 or MCC950 attenuated cognitive deficits (Squillace et al., 2022). Pharmacological treatment began 48 h before the first cisplatin dose, supporting prevention rather than reversal of established impairment. Supplementary controls did not identify corresponding impairments in locomotor activity or escape responses. These findings strengthen the case for involvement of Toll-like receptor 4 (TLR4) and NLRP3 in CICI but do not establish intestinal LPS as the upstream signal or demonstrate efficacy through a clinically practical administration route.

Host-target engagement. TLR4-associated signaling and inflammasome activation should be demonstrated in the relevant peripheral or central cell population. Nuclear factor κB (NF-κB)-dependent priming must be distinguished from subsequent NLRP3 inflammasome activation; increased NLRP3 expression alone is insufficient evidence of functional activation (Bauernfeind et al., 2009). Appropriate readouts include pathway-dependent signaling, caspase-1 processing and mature cytokine release, interpreted alongside selective pharmacological or genetic interventions. A reduction in circulating cytokines alone does not establish engagement of a particular central target.

Oncological compatibility. Suppressing microbial sensing or inflammatory signaling may also modify antimicrobial defense and cancer-treatment responses. Cognitive outcomes should therefore be assessed together with infection, neutropenia, chemotherapy exposure and tumor control. Overall, chemotherapy-specific evidence supports inflammatory mechanisms and intestinal–brain associations, whereas the source, temporal sequence and selective cognitive contribution of translocated microbial products remain incompletely established. The measurement strategy below is intended to close these gaps.

### 3.5 Cognitive domains and interpretation of behavioral endpoints

CICI should be evaluated as a set of potentially dissociable cognitive changes rather than a uniform deficit. Clinical assessment should distinguish learning and memory, working memory, executive function, attention and processing speed, using pretreatment measurements and repeated testing with consideration of practice effects and relevant normative data (Wefel et al., 2011). Patient-reported difficulties provide complementary information but should not substitute for objective domain-specific outcomes. In the breast cancer probiotic trial, benefits occurred in selected memory, visuospatial and verbal-fluency measures rather than across every cognitive test, illustrating the importance of reporting the pattern of benefit (Juan et al., 2022).

Current metabolite evidence supports only a partial mapping onto these domains. Cerebral KYNA changes were investigated alongside delayed object recognition in cisplatin-treated rats, whereas IPA supplementation improved spatial learning and memory-related water-maze outcomes after arsenic trioxide (Ren et al., 2026; Dorcas et al., 2024). These findings do not establish a kynurenine-specific executive deficit or an IPA effect across all memory domains. SCFA-associated barrier and microglial mechanisms remain plausible contributors to cognition, but the cited chemotherapy studies primarily assessed intestinal, affective or neuroimmune outcomes. Bile acid–synaptic findings from aging models likewise remain indirect evidence for CICI. Hippocampal and prefrontal mechanisms should therefore be treated as overlapping hypotheses unless domain-specific behavior, tissue exposure and selective pathway manipulation are examined together.

Behavioral tasks must be interpreted according to their implementation. Novel object recognition assesses recognition memory over a specified retention interval, whereas the standard hidden-platform Morris water maze primarily examines spatial learning and reference memory. T-maze protocols can assess different constructs: the cisplatin study used learned shock avoidance and delayed retention, rather than spontaneous alternation as a working-memory measure (Squillace et al., 2022). Delayed non-matching-to- sample, Y-maze spontaneous alternation and attentional set-shifting provide complementary assessments. In Chen and Johnston (2026), oxaliplatin alone did not impair operant delayed non-matching- to-sample performance, whereas a tested oxaliplatin plus 5-FU regimen impaired Y-maze alternation and set-shifting without significant differences in arm-entry counts, reversal learning or object recognition. These findings remain task- and regimen-specific, with treatment-related attrition warranting caution.

Reliable interpretation requires task-specific controls, summarized in Table 1. Reduced object exploration, slower swimming, altered freezing or delayed reward retrieval can reflect sickness, neuropathy, visual impairment, fatigue or motivation rather than the intended cognitive construct. Water-maze latency alone is therefore insufficient, and contextual fear conditioning requires attention to baseline freezing and shock responsiveness. Locomotor testing helps characterize activity but cannot exclude every sensory or motivational confound. Accuracy, retention, errors and task completion should be analyzed alongside relevant performance controls, with assessment timing prespecified relative to treatment and acute illness. Neither routine maze latency nor open-field movement should be relabeled as clinical processing speed.

**TABLE 1:** Cognitive domains, behavioral endpoints and limits of pathway attribution in CICI.

Cognitive domain	Clinical assessment examples	Preclinical assessment	Current pathway evidence	Essential interpretation controls
Learning and recognition memory	HVLT-R; immediate and delayed recall	Novel object recognition with a defined retention interval	Cisplatin-associated cerebral KYNA changes accompanied delayed recognition deficits; NAC benefit does not establish selective metabolite mediation (Dorcas et al., 2024).	Total exploration, object preference, sensory function, activity and retention interval
Visuospatial learning and memory	BVMT-R and other visuospatial memory measures	Hidden-platform Morris water maze with acquisition and probe testing	IPA improved water-maze outcomes after arsenic trioxide; concurrent locomotor changes require consideration (Ren et al., 2026).	Swim speed, path length, floating, visual/cued performance and probe preference
Working memory	Backward digit span; letter–number sequencing	Delayed non-matching-to-sample; Y-maze spontaneous alternation	Oxaliplatin alone spared DNMTS performance; a tested oxaliplatin plus 5-FU regimen impaired Y-maze alternation. Metabolite mediation was not established (Chen and Johnston, 2026).	Delay-dependent accuracy, omissions, arm-entry counts, nutritional state, reward motivation and treatment-related attrition
Executive function and flexibility	TMT-B; verbal fluency, interpreted with speed and language demands	Attentional set-shifting; reversal learning; puzzle box	Set-shifting and reversal outcomes can dissociate. Cisplatin-related puzzle-box findings support host-pathway investigation without establishing a microbial mediator (Squillace et al., 2022; Chen and Johnston, 2026).	Initial discrimination, perseverative errors, motor demands, motivation and obstacle difficulty
Attention and processing speed	TMT-A; digit-symbol substitution; dedicated attention measures	Tasks designed to separate accuracy, response latency and omissions	A selective metabolite–processing-speed relationship is not established by the studies reviewed here.	Motor and visual function, fatigue, omissions and speed–accuracy trade-off

Clinical and animal tests sample related constructs but are not interchangeable equivalents. Task interpretation depends on the protocol, retention interval and performance requirements. Clinical assessment examples are informed by Wefel et al. (2011) and the test battery reported by Juan et al. (2022). Abbreviations: BVMT-R, Brief Visuospatial Memory Test–Revised; DNMTS, delayed non-matching-to-sample; 5-FU, 5-fluorouracil; HVLT-R, Hopkins Verbal Learning Test–Revised; IPA, indole-3-propionic acid; KYNA, kynurenic acid; NAC, N-acetylcysteine; TMT, trail making test.

## 4 Pharmacological target validation and translational constraints

A post-chemotherapy change in abundance is a starting point, not target validation. A candidate must show a reproducible relationship with cognition, precede decline, alter cognition when selectively manipulated, engage a defined host pathway at relevant exposure and remain safe without weakening anticancer treatment. Two complementary approaches can provide this evidence: restoring an upstream microbial function and directly probing the host pathway that responds to the microbial signal. Table 2 summarizes the evidence and remaining validation gaps across these pathways.

**TABLE 2:** Evidence and validation status of candidate gut–immune–brain pathways in CICI.

Pathway or intervention	CICI-specific findings	Temporal sequence	Selective causal evidence	Host-target engagement	Oncological compatibility
SCFAs (Cristiano et al., 2022; Cross et al., 2024)	Paclitaxel: intestinal/affective outcomes; 5-FU: glial changes. Cognitive rescue was not assessed	SCFA abnormality preceding cognitive decline not established	Butyrate dosing tested; fiber changes multiple signals. Selective cognitive mediation untested	FFAR2/3, HCAR2 and HDAC mechanisms are plausible; CICI target dependence untested	Concurrent cognitive benefit, defined target engagement and preserved tumor control not established
Microbial indoles IPA example (Ren et al., 2026)	IPA improved water-maze outcomes after arsenic trioxide; locomotor effects require consideration	Patient plasma IPA reduction reported; prospective IPA–cognition sequence not established	Supplementation supports intervention benefit; endogenous microbial production loss and rescue not established	CETSA supported cellular BRD4 engagement; knockdown attenuated protection; behavioral BRD4 dependence *in vivo* remains untested	Preserved anticancer efficacy during IPA-associated cognitive protection not established
Host kynurenine pathway (Dorcas et al., 2024; Xie et al., 2026; Long et al., 2019)	Cisplatin: cerebral KYNA and recognition deficits. Oxaliplatin: intestinal IDO1/KYN findings without cognition	Tissue-specific pathway change preceding cognitive decline not established	NAC improved KYNA and recognition outcomes; pleiotropy prevents selective attribution	Host IDO1/TDO and downstream branch balance require distinction; selective cognitive dependence unresolved	Potential immune/cognitive benefit requires joint testing; IDO1 antitumor efficacy cannot be assumed
Microbially modified bile acids (Loman et al., 2024; Ren et al., 2024)	Paclitaxel altered bile acid handling and ileal *Slc10a2* expression; cognition untested. Aging evidence is indirect	Chemotherapy-specific bile acid change preceding cognitive decline not established	Selective bile acid, ASBT or receptor manipulation with CICI rescue not established in cited studies	Intestinal/immune FXR and TGR5 biology supports plausibility; CICI engagement and dependence unresolved	Concurrent cognition, tumor control, chemotherapy exposure and hepatic effects require evaluation
LPS and inflammatory signaling (Loman et al., 2019; Grant et al., 2023; Squillace et al., 2022)	Paclitaxel: gut–memory associations. Cisplatin: TLR4/NLRP3 intervention evidence; intestinal LPS source unproven	Complete barrier–exposure–neuroinflammation–cognitive decline sequence not established	Host-pathway blockade supports involvement; selective mediation by gut-derived products remains unproven	TLR4/NLRP3 involvement supported; PLX5622 findings may include peripheral CSF1R effects	Infection, antimicrobial defense and tumor response must accompany cognitive assessment
Microbiota-directed intervention Probiotic RCT (Juan et al., 2022)	Single-center breast cancer RCT reported selected cognitive benefits from the tested preparation	Intervention preceded assessment; mediation by a preceding metabolite change remains unresolved	Randomization supports the preparation; no individual microbial metabolite established as mediator	Metabolite/LTP findings lack a defined host target and behavioral metabolite rescue	No significant survival differences through 2.5 years; oncological equivalence not established

Status refers to the studies discussed in this review and to the specific agent, model and endpoint tested. Temporal validation requires a pathway abnormality to precede cognitive decline; starting an intervention before testing does not establish this sequence. “Not established” denotes an evidence gap, not evidence of no effect. Microbial indoles and host kynurenines are separated by origin; LPS is a structural product, and a probiotic preparation is an intervention rather than a metabolite. Abbreviations: 5-FU, 5-fluorouracil; ASBT, apical sodium-dependent bile acid transporter; BRD4, bromodomain-containing protein 4; CETSA, cellular thermal shift assay; CICI, chemotherapy- induced cognitive impairment; CSF1R, colony-stimulating factor 1 receptor; FFAR2/3, free fatty acid receptors 2/3; FXR, farnesoid X receptor; HCAR2, hydroxycarboxylic acid receptor 2; HDAC, histone deacetylase; IDO1, indoleamine 2,3-dioxygenase 1; IPA, indole-3-propionic acid; KYN, kynurenine; KYNA, kynurenic acid; LPS, lipopolysaccharide; LTP, long-term potentiation; NAC, N- acetylcysteine; NLRP3, NLR family pyrin domain containing 3; RCT, randomized controlled trial; SCFA, short-chain fatty acid; TDO, tryptophan 2,3-dioxygenase; TGR5, Takeda G protein-coupled receptor 5; TLR4, Toll-like receptor 4.

In practice, the target-validation package should address five linked questions. Is the abnormality reproducibly associated with domain-specific cognitive change under the intended chemotherapy regimen? Does it precede cognitive decline? Does selective correction or blockade alter cognition? Does the intervention engage the proposed host target in the relevant compartment at an achievable exposure? Does it preserve tumor control while avoiding unacceptable infection or metabolic toxicity?

### 4.1 Microbiota-directed restoration of metabolic function

Microbiota-directed interventions should be selected according to the metabolic function they are intended to restore. Probiotics deliver live microorganisms, whereas prebiotic substrates aim to support beneficial functions of the resident microbiota; synbiotic approaches combine microbial and substrate components. Evaluation should connect product composition and dosing to a measured metabolic change, cognitive benefit and mechanistic validation. A taxonomic shift alone does not establish restoration of the intended function.

Clinical evidence includes a single-center, randomized, double-blind, placebo-controlled trial in patients with stage I–III breast cancer receiving adjuvant chemotherapy (Juan et al., 2022). Of 162 randomized participants, 159 completed the study. The BIFICO preparation contained *Bifidobacterium longu*m, L*actobacillus acidophilus* and *Enterococcus faecalis*; the reported administration schedule was three capsules twice daily throughout chemotherapy. Participants received epirubicin plus cyclophosphamide, that combination with docetaxel, or docetaxel plus cyclophosphamide. The protocol assessed change from pre-chemotherapy performance, defining mild impairment as a decline of ≥1 standard deviation in at least two test items and moderate impairment as a decline of ≥2 standard deviations. Among the 159 participants who completed the study, the incidence at 21 days after the final chemotherapy cycle was 28/80 (35%) in the probiotic group versus 64/79 (81%) in the placebo group (Juan et al., 2022). Benefits were observed in selected verbal and visuospatial memory measures, line orientation and verbal fluency, rather than across every cognitive test. These findings support short-term benefit from the tested preparation, with durability requiring longer cognitive follow-up.

The metabolic findings provide a candidate mechanism rather than definitive mediation. Plasma metabolomics in a subgroup of 20 participants per arm identified nine metabolites with differential changes, including p-mentha-1,8-dien-7-ol, whose change was associated with cognitive impairment occurrence. In accompanying doxorubicin-treated rat experiments, intraperitoneal administration of this compound attenuated hippocampal long-term potentiation deficits, synaptic abnormalities, oxidative stress and glial activation (Juan et al., 2022). These endpoints support biological activity but do not establish behavioral cognitive rescue, oral bioavailability, a defined host target, or microbial production of the compound in patients. The trial therefore does not validate SCFAs, or any other individual metabolite, as the mediator of probiotic benefit.

Probiotic effects require formulation-specific evaluation. Future reports should specify strain identifiers, viable dose, treatment duration, product stability, concurrent antibiotics and adherence, together with the intended metabolic output. In the breast cancer trial, the authors reported no measurable probiotic-associated adverse effects and no statistically significant between-group differences in disease-free survival or mortality at follow-up time points through 2.5 years (Juan et al., 2022). These observations provide preliminary reassurance but do not establish oncological equivalence or exclude uncommon infections. Product and outcome specificity also matter: a phase III trial of a *Bifidobacterium* BB-12 and *Lactobacillus rhamnosus* GG mixture did not meet its primary endpoint of preventing severe irinotecan-associated diarrhea; cognition was not assessed (Mego et al., 2023).

Prebiotic and dietary approaches should be evaluated as substrate-specific interventions rather than interchangeable means of increasing SCFAs. In the 5-FU study, mice received a modified high- amylose starch diet beginning 8 days before a single intraperitoneal 5-FU dose and continuing until 16 days afterward (Cross et al., 2024). The intervention increased cecal propionate and reduced GFAP staining in selected brain regions, but did not demonstrate cognitive benefit or selective propionate mediation (Cross et al., 2024). Its pre-exposure design leaves efficacy against established cognitive impairment unresolved. Future comparisons should specify substrate composition, dose, fermentability and gastrointestinal tolerance, quantify food intake, and use intake-matched controls when appropriate. When a proposed synbiotic mechanism depends on substrate utilization by the co-administered microorganism, studies should verify that interaction and the intended metabolic output.

Defined metabolites offer direct control over administered dose, although formulation, intestinal utilization and hepatic metabolism determine target-tissue exposure. Purified metabolites alone should not be classified as postbiotics under the consensus definition of the International Scientific Association for Probiotics and Prebiotics (Salminen et al., 2021). Development should establish exposure–response relationships and engagement of the proposed host pathway: gut-directed delivery may suit an intestinal mechanism, whereas a direct central mechanism requires adequate central exposure. Across intervention types, repeated clinical measurements and mediation analyses should be complemented by selective metabolite rescue and pathway-disruption experiments, with cognitive outcomes and anticancer efficacy assessed together.

### 4.2 Host-directed validation and compartmental sampling

Host-directed experiments should link a candidate signal to its proposed cellular target. Relevant probes include SCFA receptor modulators, ligand-specific AhR interventions, IDO1 inhibitors, FXR/TGR5 modulators and interventions targeting TLR4 or NLRP3. These approaches test different mechanisms and should not be treated as interchangeable anti-inflammatory strategies. A metabolite- associated cognitive benefit should be paired with an appropriate pharmacodynamic response and a selective loss-of-function experiment, including controls for the effects of pathway disruption itself on cognition, general health and chemotherapy tolerance.

For translocation studies, an endotoxin assay should be combined with complementary host- response and epithelial-injury measurements. The Limulus amebocyte lysate (LAL) assay measures endotoxin activity, whereas validated liquid chromatography–tandem mass spectrometry (LC-MS/MS) methods can quantify selected lipid A-associated chemical markers, such as esterified 3- hydroxymyristate after appropriate processing. These measurements can diverge because plasma components can neutralize LPS activity without eliminating its chemical signal (Pais de Barros et al., 2015). LBP, soluble cluster of differentiation 14 (sCD14) and intestinal fatty acid-binding protein (I-FABP) add information about host responses and enterocyte injury, but are not interchangeable measures of permeability or proof of gut-derived translocation (Tudesq et al., 2017).

Analytical reporting should specify collection materials, storage conditions, detection and quantification limits, process blanks and matrix-specific validation. LAL testing requires checks for inhibition or enhancement, including dilution and spike-recovery experiments; LC-MS/MS requires suitable internal standards, recovery assessment and calibration appropriate to the measured chemical species. Functional permeability testing and intestinal histology should accompany circulating markers where feasible. Concordant findings strengthen interpretation, but gut-source attribution additionally requires approaches such as tracing or selective barrier manipulation. Infection at other sites and altered hepatic or renal clearance must also be considered.

Animal studies should prioritize paired portal-vein and systemic blood sampling with cerebrospinal fluid (CSF) or brain tissue at matched time points when central exposure is proposed. Intestinal contents and mucosal tissue provide complementary measures of luminal availability and local responses. This design helps distinguish intestinal availability, absorption, hepatic first-pass handling and central penetration, although concentration differences alone do not quantify production or clearance. Sampling should be standardized to dosing and feeding, with controls for blood contamination of CSF and residual vascular signal in brain tissue. Matched terminal cohorts may be needed for invasive sampling. An intestinal or peripheral immune mechanism does not require the parent molecule to enter the brain.

Target validation should finally integrate exposure, tissue-specific signaling, domain-specific cognitive outcomes and tumor response under the intended chemotherapy regimen. Behavioral controls should address motor impairment, sickness and altered motivation. Anti-inflammatory or antioxidant effects become mechanistically informative when connected to a defined exposure–target–cognition relationship; their coexistence alone does not validate a microbial metabolite or translocation pathway.

### 4.3 Oncological compatibility as a prerequisite for target validation

The microbiota can influence chemotherapy biotransformation, exposure, mucosal recovery and antitumor immunity (Alexander et al., 2017; Böhm et al., 2025). Pharmacokinetics and tumor response should be prespecified as key translational endpoints in studies of cognitive protection. Antibiotics, proton-pump inhibitors, corticosteroids, diet, cancer type and regimen can also modify microbial metabolism and treatment response, so prospective recording and appropriate stratification are essential.

This concern is mechanistically concrete rather than hypothetical. Microbial enzymes can modify drug structure, alter enterohepatic recirculation and change the balance between active and inactive metabolites. Microbial depletion can also change mucosal repair and immune surveillance, thereby affecting the therapeutic index through more than one route (Alexander et al., 2017). For this reason, a metabolite-directed neuroprotective strategy should be evaluated with the same pharmacological discipline as a drug–drug interaction study: characterize exposure, active metabolites, tumor response and toxicity under the intended chemotherapy schedule.

Infectious safety is particularly relevant during mucosal injury and neutropenia. Probiotic safety data are strain- and population-specific, and rare bloodstream infections related to probiotic organisms have occurred in vulnerable patients (Doron and Snydman, 2015; Yelin et al., 2019). Fecal microbiota transplantation adds risks of transmitting pathogens and resistance determinants; donor-derived extended-spectrum β-lactamase-producing *Escherichia coli* bacteremia has been reported (DeFilipp et al., 2019). Defined metabolites, analogues and non-viable products may reduce infectious risk, but still require systemic and tumor-specific evaluation. These constraints favor a staged, biomarker-guided pathway from reproducible abnormality to causal manipulation, host-target engagement and only then clinical testing.

Risk may not be uniform across the treatment course. Neutropenia, mucosal barrier injury, central venous access and broad-spectrum antibiotic exposure can create periods in which live organisms are less appropriate, even if they are well tolerated later (Doron and Snydman, 2015). Product characterization, lot testing, strain-level identification and predefined stopping criteria should be incorporated from the first clinical study. Non- viable organisms and defined metabolites may be preferable for high-risk patients, but they should not be assumed to be safer until systemic inflammatory and metabolic effects have been measured.

## 5 Discussion and future directions

The studies reviewed place the candidate pathways at different stages of validation. IPA supplementation provides cognitive intervention evidence in an arsenic trioxide model, whereas cerebral KYNA changes after cisplatin identify a host metabolic branch for selective testing (Ren et al., 2026; Dorcas et al., 2024). The cited SCFA and bile acid chemotherapy studies chiefly document intestinal, affective, metabolic or neuroimmune effects. Clinical microbiome associations and the preparation-specific probiotic trial extend the rationale to patients, while individual metabolite mediation remains unresolved (Otto-Dobos et al., 2024; Cross et al., 2026; Juan et al., 2022).

A metabolite-centered framework makes these differences experimentally useful. SCFA receptor and barrier biology provides tractable mechanisms for testing (Dalile et al., 2019; Fock and Parnova, 2023), whereas tryptophan pathways require greater resolution of metabolic flux and tissue compartments (Kennedy et al., 2017). Bile acid pharmacology must be linked to the enterohepatic changes produced by the chemotherapy regimen (Loman et al., 2024), and inflammatory products should be evaluated as modifiers of metabolite signaling (Ghosh et al., 2020). The next priorities are to test IPA target dependence *in vivo* while measuring tumor control, selectively manipulate the implicated kynurenine branch, and establish cognitive effects of defined SCFA and bile acid interventions under specific chemotherapy regimens.

The next studies should be designed around biological sequence. Paired fecal and plasma metabolomics before, during and after chemotherapy should accompany standardized objective and patient-reported cognitive assessments. Measures of permeability, inflammation and neural injury can locate intermediate steps. Sampling should precede treatment, capture the period of intestinal toxicity and continue after chemotherapy, because a metabolite that follows rather than precedes cognitive change may be a consequence or recovery marker. Candidates emerging from these cohorts should then undergo selective supplementation, depletion, enzyme inhibition or receptor blockade in tumor- bearing models. Dose–response, measured exposure and loss of effect after pathway disruption would provide stronger evidence than compositional microbiome changes.

Preclinical work should also reproduce the clinical context as closely as possible. Models should use clinically relevant drug schedules, include both sexes where appropriate, and measure food intake, weight, sickness behavior and peripheral neuropathy so that cognition is not interpreted in isolation. Germ-free, antibiotic-depleted or defined-community models can help establish microbial dependence, but they should be followed by rescue experiments with a metabolite or selective host probe. Replication across more than one chemotherapy agent would indicate whether the pathway is a general CICI mechanism or specific to one regimen.

Clinical trials should enroll or stratify patients by the metabolic abnormality being targeted and include pharmacokinetic and target-engagement endpoints. An unselected trial may dilute benefit if only a subgroup has the relevant deficiency or pathway activation. Early studies should establish biological activity and exposure before being powered for cognitive efficacy, with an analysis plan that distinguishes prevention from treatment of established symptoms. Tumor response, chemotherapy exposure, infection, survival-related outcomes, antibiotic use, diet, baseline cognition and regimen must be captured prospectively. This design shifts the field from descriptive dysbiosis toward mechanism-based patient selection.

A staged clinical program can reduce avoidable ambiguity. An observational phase can define the metabolic phenotype and its temporal relationship to cognition; a small mechanistic trial can test exposure and target engagement; and a larger efficacy trial can then enroll the subgroup most likely to respond. Cognitive outcomes should be analyzed alongside patient-reported function, because a statistically significant test-score change may not translate into meaningful daily benefit (Wefel et al., 2011; Ahles and Root, 2018). This sequence also creates an opportunity to stop development early if the intervention changes chemotherapy exposure or tumor outcomes in an unfavorable direction.

In conclusion, microbial metabolites are plausible mediators of communication between chemotherapy-injured intestine and brain, but they are not yet validated therapies for CICI. Their translational value will depend on CICI-specific temporal evidence, selective causal manipulation, demonstrable host-target engagement and preserved anticancer efficacy. Meeting these requirements would convert promising associations into testable precision-pharmacology strategies.
